# 基于^18^F-FDG PET/CT代谢参数构建非小细胞肺癌PD-L1表达的列线图预测模型

**DOI:** 10.3779/j.issn.1009-3419.2023.101.32

**Published:** 2023-11-20

**Authors:** Luoluo HAO, Lifeng WANG, Mengyao ZHANG, Jiaming YAN, Feifei ZHANG

**Affiliations:** ^1^014040 包头，内蒙古科技大学包头医学院研究生院; ^1^Baotou Medical College, Inner Mongolia University of Science & Technology, Baotou 014040, China; ^2^710600 西安，核工业四一七医院; ^2^Nuclear Industry 417 Hospital, Xi’an 710600, China; ^3^010020 呼和浩特，内蒙古自治区人民医院; ^3^Inner Mongolia People's Hospital, Hohhot 010020, China

**Keywords:** 肺肿瘤, ^18^F-脱氧葡萄糖正电子发射计算机断层扫描, 代谢参数, 程序性细胞死亡配体1, 列线图, Lung neoplasms, ^18^F-FDG PET/CT, Metabolic parameters, Programmed cell death ligand 1, Nomogram

## Abstract

**背景与目的:**

近年来，程序性细胞死亡受体1（programmed cell death 1, PD-1）/程序性细胞死亡配体1（programmed cell death ligand 1, PD-L1）免疫抑制剂为代表的免疫疗法很大程度地改变了非小细胞肺癌（non-small cell lung cancer, NSCLC）的治疗现状。目前PD-L1已经成为了筛选NSCLC免疫治疗获益人群的重要生物标志物，但是如何便捷且准确地检测NSCLC患者PD-L1是否表达是困扰临床医师的难题。本研究旨在基于^18^F-脱氧葡萄糖（^18^F-fluorodeoxy glucose, ^18^F-FDG）正电子发射计算机断层扫描（positron emission tomography/computed tomography, PET/CT）代谢参数构建NSCLC患者PD-L1表达的列线图预测模型并评估其预测价值。

**方法:**

回顾性收集2016年9月至2021年7月内蒙古自治区人民医院收治的155例NSCLC患者的^18^F-FDG PET/CT代谢参数、临床病理资料及PD-L1检测结果。将患者分为训练组（n=117）及内部验证组（n=38），按照同样的标准另收集本院2021年8月至2022年7月NSCLC患者51例作为外部验证组。然后均根据PD-L1检测结果分为PD-L1+组与PD-L1-组。对训练组患者的代谢参数及临床病理资料进行单因素及二元Logistic回归分析，基于筛选出的独立影响因素构建列线图预测模型。在训练组及内外部验证组中均通过受试者工作特征（receiver operating characteristic, ROC）曲线、校准曲线及临床决策曲线（decision curve analysis, DCA）来评估列线图模型效果。

**结果:**

二元Logistic回归分析表明，肿瘤代谢体积（metabolic tumor volume, MTV）、性别及肿瘤直径是PD-L1表达的独立影响因素，然后基于上述独立影响因素构建列线图预测模型。模型在训练组中的ROC曲线显示，曲线下面积（area under the curve, AUC）为0.769（95%CI: 0.683-0.856），最佳截断值为0.538。内部验证组的AUC为0.775（95%CI: 0.614-0.936），外部验证组的AUC为0.752（95%CI: 0.612-0.893）。校准曲线经Hosmer-Lemeshow检验结果显示，训练组（χ^2^=0.040, P=0.979）、内部验证组（χ^2^=2.605, P=0.271）及外部验证组（χ^2^=0.396, P=0.820）均具有良好的校准度。DCA曲线显示，模型在较大的阈值范围内（训练组：0.00-0.72，内部验证组：0.00-0.87，外部验证组：0.00-0.66）能使患者临床获益。

**结论:**

基于^18^F-FDG PET/CT代谢参数构建的列线图预测模型在预测NSCLC患者PD-L1表达中有较大的应用价值。

## Abstract (en)

**Background and objective:**

In recent years, immunotherapy represented by programmed cell death 1 (PD-1)/programmed cell death ligand 1 (PD-L1) immunosuppressants has greatly changed the status of non-small cell lung cancer (NSCLC) treatment. PD-L1 has become an important biomarker for screening NSCLC immunotherapy beneficiaries, but how to easily and accurately detect whether PD-L1 is expressed in NSCLC patients is a difficult problem for clinicians. The aim of this study was to construct a Nomogram prediction model of PD-L1 expression in NSCLC patients based on ^18^F-fluorodeoxy glucose (^18^F-FDG) positron emission tomography/conputed tomography (PET/CT) metabolic parameters and to evaluate its predictive value.

**Methods:**

Retrospective collection of ^18^F-FDG PET/CT metabolic parameters, clinicopathological information and PD-L1 test results of 155 NSCLC patients from Inner Mongolia People's Hospital between September 2016 and July 2021. The patients were divided into the training group (n=117) and the internal validation group (n=38), and another 51 cases of NSCLC patients in our hospital between August 2021 and July 2022 were collected as the external validation group according to the same criteria. Then all of them were categorized according to the results of PD-L1 assay into PD-L1+ group and PD-L1- group. The metabolic parameters and clinicopathological information of patients in the training group were analyzed by univariate and binary Logistic regression, and a Nomogram prediction model was constructed based on the screened independent influencing factors. The effect of the model was evaluated by receiver operating characteristic (ROC) curve, calibration curve and decision curve analysis (DCA) in both the training group and the internal and external validation groups.

**Results:**

Binary Logistic regression analysis showed that metabolic tumor volume (MTV), gender and tumor diameter were independent influences on PD-L1 expression. Then a Nomogram prediction model was constructed based on the above independent influences. The ROC curve for the model in the training group shows an area under the curve (AUC) of 0.769 (95%CI: 0.683-0.856) with an optimal cutoff value of 0.538. The AUC was 0.775 (95%CI: 0.614-0.936) in the internal validation group and 0.752 (95%CI: 0.612-0.893) in the external validation group. The calibration curves were tested by the Hosmer-Lemeshow test and showed that the training group (χ^2^=0.040, P=0.979), the internal validation group (χ^2^=2.605, P=0.271), and the external validation group (χ^2^=0.396, P=0.820) were well calibrated. The DCA curves show that the model provides clinical benefit to patients over a wide range of thresholds (training group: 0.00-0.72, internal validation group: 0.00-0.87, external validation group: 0.00-0.66).

**Conclusion:**

The Nomogram prediction model constructed on the basis of ^18^F-FDG PET/CT metabolic parameters has greater application value in predicting PD-L1 expression in NSCLC patients.

肺癌处于恶性肿瘤死亡率的前列，非小细胞肺癌（non-small cell lung cancer, NSCLC）是其最主要的病理类型，约占85%^[1]^。NSCLC由于起病隐蔽，超过50%的患者首次到医院就诊时疾病已经进展到晚期甚至出现远处转移^[2]^。尽管现在化疗、放疗和靶向药物等NSCLC的治疗方法有所发展，但患者5年生存率仍然很低^[3]^。近年来以程序性细胞死亡受体1（programmed cell death 1, PD-1）/程序性细胞死亡配体1（programmed cell death ligand 1, PD-L1）免疫抑制剂为代表的免疫疗法使NSCLC患者的治疗发生了里程碑式的变化^[4]^。PD-1/PD-L1检查点抑制剂可以特异性阻断PD-1与PD-L1的聚合，重新激活被抑制的T细胞，实现抗肿瘤作用。2019年一项发表于柳叶刀杂志的KEYNOTE-042研究^[5]^表明，在PD-L1表达阳性即肿瘤细胞阳性比例评分（tumor proportion score, TPS）≥1%的晚期NSCLC患者中，PD-1抑制剂（帕博利珠单抗）单药治疗组相比于含铂双药化疗一线治疗组可以显著延长总生存期。目前美国食品药品监督管理局（Food and Drug Administration, FDA）已批准帕博利珠单抗单独作为有PD-L1表达（TPS≥1%）的晚期患者的一线单药治疗^[6]^。此外还有多项研究^[7,8]^表明在不同的PD-L1表达水平下，晚期NSCLC的一线治疗中帕博利珠单抗联合化疗均可表现出显著的生存获益。近期中华医学会肺癌临床诊疗指南（2023版）^[9]^更新内容中指出，以PD-1/PD-L1检查点抑制剂为主的新辅助免疫治疗在早中期NSCLC患者中也可以显著改善预后。因此，有必要在治疗前明确NSCLC患者是否存在PD-L1表达。目前基于活组织检查的（immunohistochemistry, IHC）法检测PD-L1表达状态因有创及样本量等因素具有一定的局限性^[10⇓-12]^。这给NSCLC患者的个体精准化治疗带来困难，临床上迫切需要新的指标来预测PD-L1是否表达。正电子发射计算机断层扫描（positron emission tomography/computed tomography, PET/CT）可以定量分析代谢参数的变化，从分子影像水平反映肿瘤组织的早期变化，从而对肺癌进行诊断和预后评估。其中，^18^F-脱氧葡萄糖（^18^F-fluorodeoxy glucose, ^18^F-FDG）是临床中应用最多的PET/CT显影剂，已有研究^[13,14]^证明，肿瘤细胞对^18^F-FDG的摄取与葡萄糖转运蛋白-1（glucose transporter-1, Glut-1）和缺氧微环境密切相关，而Glut-1和缺氧微环境是影响肿瘤PD-L1表达的重要因素^[15,16]^。因此应用^18^F-FDG PET/CT代谢参数预测NSCLC患者PD-L1表达成为可能。

列线图模型因其能够量化影响因素且简单、便捷、可视化等特性已经广泛应用于临床预测研究。目前尚无研究基于^18^F-FDG PET/CT代谢参数构建预测NSCLC患者PD-L1表达的列线图模型。为此本研究旨在通过分析代谢参数最大标准摄取值（maximum standardized uptake value, SUVmax）、肿瘤代谢体积（metabolic tumor volume, MTV）、糖酵解总量（total lesion glycolysis, TLG）及临床病理特征与NSCLC患者PD-L1表达的关联，筛选出PD-L1表达的独立影响因素，建立一个基于^18^F-FDG PET/CT代谢参数的预测NSCLC患者PD-L1表达的列线图模型并评估其预测价值。

## 1 资料与方法

### 1.1 研究对象与分组

本研究回顾性收集2016年9月至2021年7月在内蒙古自治区人民医院接受^18^F-FDG PET/CT检查的155例NSCLC患者，将患者按3:1的比例划分（采用R3.4.2软件）为训练组（117例）和内部验证组（38例），另收集本院2021年8月至2022年7月51例NSCLC患者作为外部验证组。纳入标准：（1）经手术或穿刺活检病理证实为原发性腺癌（adenocarcinoma, ADC）或鳞癌（squamous cell carcinoma, SCC）；（2）手术或活检前进行^18^F-FDG PET/CT检查；（3）IHC法检测到PD-L1表达水平；（4）无其他恶性肿瘤病史。排除标准：（1）在^18^F-FDG PET/CT扫描和IHC法检测前接受过抗肿瘤治疗；（2）原发灶肿瘤直径<1 cm（避免部分容积效应影响）；（3）图像上未发现^18^F-FDG摄取；（4）临床资料缺失。

查阅病历，收集患者的临床病理特征资料，如性别、吸烟史、肿瘤直径、血清癌胚抗原（carcinoembryonic antigen, CEA）水平、肿瘤分期、病理类型及表皮生长因子受体（epidermal growth factor receptor, EGFR）状态。

### 1.2 ^18^F-FDG PET/CT扫描与代谢参数测量

患者的^18^F-FDG PET/CT检查均在GE Discovery Elite PET/CT设备上进行。^18^F-FDG（3.7-4.44 MBq/kg，放化学纯度>95%）静脉注射前禁食6-8 h，血糖<11 mmol/L，注射后约1 h开始进行图像采集。首先，使用全身低剂量CT扫描（管电流150 mA，管电压120 kV，层厚3.75 mm）进行衰减校正，随后行PET扫描（每个床位3 min，7-10个床位）。扫描范围：颅顶至大腿根部，采集结束后使用有序子集最大期望法（ordered subset expectation maximization, OSEM）重建PET图像，然后在Xeleris工作站进行图像融合，最后由机器自动勾画感兴趣区（region of interest, ROI）并获得病灶的SUVmax，然后设置SUVmax的40%为阈值得到MTV及平均标准摄取值（average standardized uptake value, SUVavg），由MTV×SUVavg获得TLG。

### 1.3 PD-L1表达状态的检测

使用Vantana公司的试剂盒（SP142）进行PD-L1检测。TPS是指在至少含有100个活的癌细胞巢中，阳性癌细胞数（部分或者完整细胞膜染色）占样本中全部癌细胞的百分比。按照TPS评分将本研究中患者的PD-L1检测情况分为PD-L1+组（TPS≥1%）和PD-L1-组（TPS<1%）。

### 1.4 统计学分析和模型建立

利用SPSS 25.0和R3.4.2软件进行统计分析。利用R软件包“car”与“survival”按照3:1随机划分训练组和内部验证组。在训练组的单因素分析中，非正态分布的计量资料以中位数和四分位间距[M（Q1,Q3）]表示，采用Mann-Whitney U检验，如年龄及SUVmax、MTV、TLG；计数资料以例数描述，采用χ^2^检验，如性别、吸烟史、肿瘤直径、CEA、肿瘤分期、病理类型及EGFR。然后将有统计学差异的变量纳入二元Logistic回归分析，筛选出PD-L1表达的独立影响因素，检验水准α=0.05。采用R软件包“rms”根据独立影响因素构建列线图模型；绘制受试者工作特征（receiver operating characteristic, ROC）曲线评估模型的区分度；绘制校准曲线并通过Hosmer-Lemeshow检验评价预测模型的校准度；绘制决策曲线（decision curve analysis, DCA）评价模型的临床净获益；在内外部验证组中对模型效果进行验证。

## 2 结果

### 2.1 患者的临床资料

训练组117例患者中，PD-L1+组57例（48.7%），PD-L1-组60例（51.3%））；内部验证组38例患者中，PD-L1+组18例（47.3%），PD-L1-组20例（52.7%）；外部验证组51例患者中，PD-L1+组21例（41.1%），PD-L1-组30例（58.9%）。

### 2.2 列线图模型的建立

在训练组中，单因素分析结果显示，患者MTV（P<0.001）、TLG（P=0.031）、性别（P=0.004）、肿瘤直径（P=0.008）与NSCLC患者PD-L1表达有关；而SUVmax（P=0.558）、吸烟史（P=0.054）、年龄（P=0.175）、CEA水平（P=0.081）、肿瘤分期（P=0.300）、病理类型（P=0.171）及EGFR（P=0.190）与PD-L1表达均无关联（表1）。将单因素分析中P<0.05的代谢参数及临床特征进一步行二元Logistic回归分析，结果显示，MTV（P=0.003）、性别（P=0.045）、肿瘤直径（P=0.008）是PD-L1表达的独立影响因素（表2）。基于筛选出来的独立影响因素利用R3.4.2软件构建列线图模型（图1），通过计算各项指标的评分之和得到相对应的PD-L1表达预测风险。

**表1:** 训练组患者PET/CT代谢参数及临床病理特征的单因素分析

Features	PD-L1+ (n=57)	PD-L1- (n=60)	χ^2^/Z	P
Age (yr) (range)	64.0 (57.0, 68.0)	68.0 (57.0, 73.0)	-1.356^a^	0.175
Gender			8.145	0.004
Male	34	20		
Female	23	40		
Smoking history			3.706	0.054
Yes	28	19		
No	29	41		
Tumor diameter (cm)			7.130	0.008
>3	34	21		
≤3	23	39		
CEA (ng/mL)			3.052	0.081
≥5	32	24		
<5	25	36		
Tumor stage			1.037	0.300
I/II	24	31		
III/IV	33	29		
Pathological type			1.876	0.171
ADC	34	43		
SCC	23	17		
EGFR			4.760	0.190
Mutant (ADC)	19	18		
Wild type (ADC)	15	25		
Mutant (SCC)	8	3		
Wild type (SCC)	15	14		
SUVmax (range)	10.70 (6.55, 15.60)	12.25 (7.25, 17.30)	-0.586^a^	0.558
MTV (range)	11.96 (8.05, 15.15)	7.46 (4.96, 9.77)	-4.038^a^	<0.001
TLG (range)	91.55 (40.00, 132.90)	50.62 (23.81, 112.02)	-2.154^a^	0.031

^a^ is the Z-value; PET/CT: positron emission tomography/computed tomography; PD-L1: programmed cell death ligand 1; CEA: carcinoembryonic antigen; ADC: adenocarcinoma; SCC: squamous cell carcinoma; EGFR: epidermal growth factor receptor; SUVmax: maximum standardized uptake value; MTV: metabolic tumor volume; TLG: total lesion glycolysis.

**表2:** 训练组患者的二元Logistic回归分析

Factor	β	SE	Wald	OR	95%CI	P
MTV	0.241	0.080	8.946	1.272	1.086-1.489	0.003
TLG	-0.007	0.005	2.287	0.993	0.984-1.002	0.130
Gender	-0.876	0.438	4.000	0.416	0.177-0.983	0.045
Tumor diameter (cm)	-1.140	0.431	6.989	0.320	0.137-0.745	0.008

β is the regression coefficient and SE is the standard error. OR: odd ratio; CI: confidence interval.

**图1:**
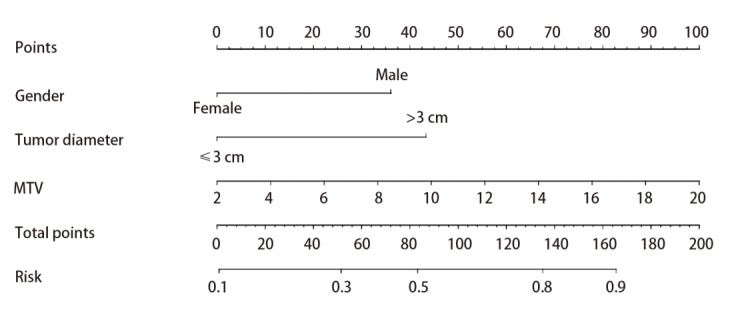
预测NSCLC患者PD-L1表达的列线图模型

### 2.3 列线图模型的评价与内外部验证

#### 2.3.1 区分度

训练组和验证组的ROC曲线显示，训练组的曲线下面积（area under the curve, AUC）为 0.769（95%CI: 0.683-0.856），最佳截断值为0.538，灵敏度为66.7%，特异度为80.0%；内部验证组的AUC为0.775（95%CI: 0.614-0.936），最佳截断值为0.458，灵敏度为83.3%，特异度为75.0%；外部验证组的AUC为0.752（95%CI: 0.612-0.893），最佳截断值为0.514，灵敏度为76.2%，特异度为73.3%（图2）。

**图2:**
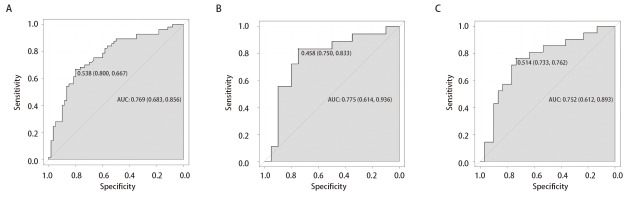
模型预测NSCLC患者PD-L1表达的ROC曲线。A：训练组；B：内部验证组；C：外部验证组。

#### 2.3.2 校准度

绘制预测模型的校准曲线图，经Hosmer-Lemeshow检验结果显示，训练组（χ^2^=0.040, P=0.979）、内部验证组（χ^2^=2.605, P=0.271）及外部验证组（χ^2^=0.396, P=0.820）均具有良好的校准度（图3）。

**图3:**
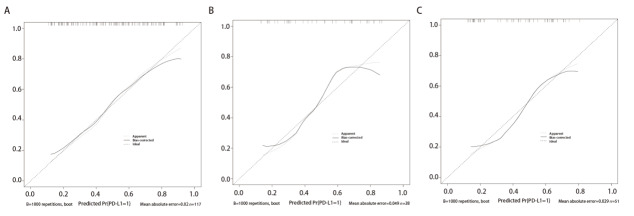
模型预测NSCLC患者PD-L1表达的校准曲线。A：训练组；B：内部验证组；C：外部验证组。

#### 2.3.3 临床获益分析

DCA曲线结果显示，在训练组中，模型在风险阈值为0.00-0.72时能为患者带来临床净获益；在内部验证组中，临床净获益的风险阈值范围为0.00-0.87；在外部验证组中，临床净获益的风险阈值范围为0.00-0.66（图4）。典型病例见图5、图6。

**图4:**
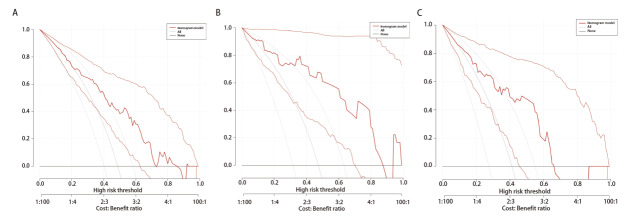
模型预测NSCLC患者PD-L1表达的DCA曲线。A：训练组；B：内部验证组；C：外部验证组。

**图5:**
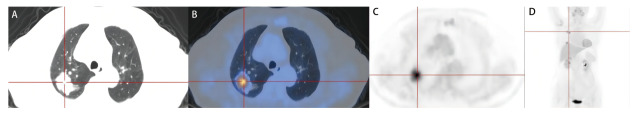
患者：男，59岁，右肺上叶肿块，病理为腺癌，SUVmax：13.70，MTV：10.01，TLG：100.98，经列线图模型计算，其总分约为124分，对应风险值高于阈值0.538，预测为PD-L1表达阳性; SP142试剂盒检测结果：PD-L1表达率TPS=5%。A：CT肺窗；B：PET/CT融合图；C：PET图；D：全身MIP图。

**图6:**
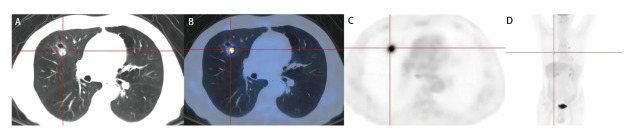
患者：男，74岁，右肺上叶结节，病理为腺癌，SUVmax：15.10，MTV：3.70，TLG：37.74，经列线图模型计算，其总分约为44分，对应风险值低于阈值0.538，预测为PD-L1表达阴性；SP142试剂盒检测结果：PD-L1表达率TPS<1%。A：CT肺窗；B：PET/CT融合图；C：PET图；D：全身MIP图。

## 3 讨论

近年来，免疫治疗的出现从根本上改变了NSCLC的治疗模式。在免疫治疗的策略中，因PD-1/PD-L1免疫抑制剂可以显著改善NSCLC患者的预后，目前在临床上应用最为广泛。最近发布的美国国立综合癌症网络（National Comprehensive Cancer Network, NCCN）指南^[17]^指出：PD-L1表达水平是唯一临床批准的筛选免疫治疗人群的生物标志物，且推荐（1类）对于转移性NSCLC患者在接受一线治疗之前，应用IHC法检验PD-L1表达情况。然而在实际的临床操作中，因为患者病情较重或拒绝有创检查、病灶位置及大小、活检样本含量不足等情况都会限制对PD-L1表达状态的评估^[10⇓-12]^。因此如何便捷地预测PD-L1的表达是目前临床NSCLC管理中的重要问题。^18^F-FDG PET/CT是当今世界上最先进的非侵入性显性技术之一，目前已有研究^[18]^利用^18^F-FDG PET/CT影像组学构建了预测NSCLC患者PD-L1表达的列线图模型，但是影像组学需要进行复杂的图像特征分割、提取及筛选，并且在无法获得分析影像组学特征所使用的机器人学习模型的核心算法及参数时，基于影像组学构建的预测模型难以在NSCLC患者管理中实现应用。相较于PET/CT影像组学特征而言，PET/CT代谢参数更容易获得，基于PET/CT代谢参数构建的列线图模型也更具有临床适用性，近年来已有研究^[19⇓-21]^表明^18^F-FDG PET/CT代谢参数与NSCLC患者PD-L1表达之间存在密切关联。因此，本研究旨在开发一个基于PET/CT代谢参数的预测NSCLC患者PD-L1表达的列线图模型，为临床上诊断NSCLC患者PD-L1是否表达提供便利，为治疗决策提供支持。

在本研究中，我们通过单因素分析发现，SUVmax（P=0.558）在PD-L1+组与PD-L1-组间差异无统计学意义，MTV（P<0.001）及TLG（P=0.031）在两组间差异有统计学意义，这与既往的研究^[22⇓-24]^结果相似。笔者认为本研究中PD-L1表达与SUVmax无关而与MTV及TLG有关的原因可能为：（1）病灶SUVmax的测量值容易受到多方面因素影响，如患者血糖水平、显像剂剂量、成像设备参数及图像获取时间；（2）本研究中入组ADC的占比较大，而ADC属于高度异质性的恶性肿瘤，可以由多种组织学结构共同组成。已有研究^[25]^表明PD-L1表达与ADC中的贴壁结构、微乳头结构及实体型结构显著相关，而SUVmax只能代表病灶局部单一像素的^18^F-FDG最高摄取值，无法反映肿瘤组织整体的代谢活性，因此可能无法准确反映PD-L1的表达状态。本研究中体积代谢参数MTV是由机器按照SUVmax的40%为阈值自动计算出肿瘤组织中^18^F-FDG摄取超过设定阈值的体积，而TLG是由SUVavg×MTV获得，根据计算方式可知MTV及TLG相较于SUVmax能体现出更多肿瘤组织的代谢特性，因此在预测PD-L1的表达状态中更具有优势。然而Jreige等^[26]^的研究发现，PD-L1表达与SUVmax、MTV、TLG均无明显相关性，这与我们的研究结果不符。考虑原因可能是：（1）该研究计算MTV的阈值为SUVmax的42%与我们的研究中的阈值不同可能会造成结论不同；（2）已有研究^[27,28]^表明，ADC与SCC的FDG摄取和代谢是有差异的，该研究中ADC与SCC的入组病例数接近，而本研究中入组的ADC病例明显较多。（3）该研究仅纳入49例NSCLC患者，其观点还需要更多大样本研究进行证实。

本研究进一步行二元Logistic回归分析表明，MTV与PD-L1表达关联仍然显著，而TLG与PD-L1表达无关。原因可能是TLG的计算公式MTV×SUVavg扩大了误差导致。本研究临床因素中，性别及肿瘤直径是PD-L1表达的独立影响因素，因而纳入列线图模型中，这与以往的研究^[19,29]^相符，即男性及更大的肿瘤更容易存在PD-L1表达。原因可能是，一般来说肿瘤直径越大则肿瘤细胞越多，更多的肿瘤细胞更容易产生缺氧微环境，而在缺氧状态下，PD-L1更容易发生表达^[30]^；目前关于PD-L1表达在性别中的差异其机制尚不清楚，还有待更多研究进一步探索。

本研究根据单因素以及二元Logistic回归分析最终筛选出的变量（MTV、性别及肿瘤直径）构建的列线图模型结果显示，预测模型在训练组及内外部验证组中ROC曲线的AUC均>0.75，提示模型具有较好的区分度，区分NSCLC患者是否存在PD-L1表达的能力较强。对预测模型的校准曲线进行Hosmer-Lemeshow检验，结果显示，在训练组及内外部验证组中Hosmer-Lemeshow检验的P值均>0.05，表明预测模型在训练组及验证组中均具有良好的校准度，预测NSCLC患者发生PD-L1表达的风险与实际发生风险具有较好的一致性。此外，本次研究还分析了模型的DCA曲线，结果显示，列线图模型在较大的阈值范围内均能为患者带来临床获益。但由于内部验证组样本量较小，导致在计算不同风险阈值下临床净获益时曲线波动较大，特别是在风险阈值高于0.7后波动比较明显。本文构建的列线图其临床使用价值体现在：（1）该模型可以把复杂的数学公式转变为可视化的图形，可以根据不同影响因素对临床事件影响程度的高低，给每个影响因素的取值水平进行赋分并计算总评分获得该个体结局事件的预测概率。（2）纳入的3个影响因素均为临床中简单易获得的指标。（3）本研究给出了列线图预测模型中PD-L1表达的风险概率阈值（0.538），可进一步提高临床医师判断NSCLC患者PD-L1是否表达的准确率。（4）本研究入组了不同肿瘤分期的NSCLC患者，根据中华医学会肺癌临床诊疗指南（2023版）发布的最新内容可知^[9]^，在驱动基因阴性且PD-L1表达阳性（TPS≥1%）的情况下，IIA-IIIA期NSCLC患者可在以铂类为基础的化疗后行PD-L1抑制剂（阿替利珠单抗）辅助治疗（2A类推荐证据），IV期NSCLC患者可以使用单药PD-1抑制剂（帕博利珠单抗）进行治疗（1类推荐证据），由此可知本研究构建的列线图模型在晚期NSCLC患者具有较大的应用价值。同时在指南中也指出，在驱动基因阴性的I/II期及可切除的III期患者中建议使用PD-1/PD-L1免疫抑制剂联合含铂双药化疗进行新辅助治疗（1类推荐证据）；在不可切除的III期患者中，建议同步化放疗后行PD-L1抑制剂（度伐利尤单抗）进行巩固治疗（1类推荐证据）。虽然指南中没有限制新辅助免疫治疗及巩固治疗中PD-L1表达情况，但有研究^[31,32]^显示免疫治疗的免疫应答率在PD-L1阳性患者中更高，因此本研究构建的列线图模型在早中期NSCLC患者中也可能具有一定的应用价值。

本研究具有局限性：（1）本研究属于单中心回顾性研究且样本量不够大。（2）PD-L1检测标本获取方式不一致。本研究中I/II期患者多为手术切除获得，III/IV期患者多为活组织穿刺获得。有研究^[33]^表明，PD-L1表达在活组织穿刺标本与手术标本间存在一定的差异。因此本研究结果可能存在一定的偏倚。（3）本研究仅采用了1种PD-L1检测试剂盒（SP142），而目前批准投入临床使用的PD-L1检测试剂盒有4种，分别为：Dako平台的22C3和28-8及Vantana平台的SP263和SP142。不同的试剂盒进行检测可能会导致结果不同^[34]^。因此，还需要进一步对比不同试剂盒的检测效果以提高结果的可信度。

总之，本研究结果显示，在NSCLC人群中，PD-L1表达与MTV、性别及肿瘤直径有关联，联合这三个因素构建的列线图模型进行综合评价有助于临床医师较早甄别PD-L1表达风险高的患者，并进行免疫治疗获益，尤其对无法进行组织活检及手术的患者更有价值。

**Competing interests**

The authors declare that they have no competing interests.
